# Homœopathy and Allopathy

**Published:** 1891-02

**Authors:** 


### HOMŒOPATHIC PHYSICIAN,

A MONTHLY JOURNAL OF

HOMŒOPATHIC MATERIA MEDICA AND CLINICAL MEDICINE.

"If our school ever gives up the strict inductive method of Hahnemann, we are lost, and deserve only to be mentioned as a caricature in the history of medicine."—CONSTANTINE HERING.

Vol. XI. FEBRUARY, 1891. No. 2.

### EDITORIAL.

HOMŒOPATHY AND ALLOPATHY are as far apart regarding the nature of disease as they are at variance in respect of its cure. The allopath pretends to know, not only the character of various affections, but in many cases he assumes to be able to determine their cause. That it is mere assumption mortality tables will testify. Not only this, he views disease as something ponderable, appreciable, a *thing* which can not only be seen, but which can be driven from the system as evil spirits were driven from man in the dark ages by various methods of exorcism. The only apparent difference between the ancient mode and the present allopathic way of treating sickness is that the latter relies on noxious drugs, while the former trusted mostly to incantations. Knowing of what harm drugs are capable we should infinitely prefer the ancient way.

The homœopathician, on the other hand, makes no pretension whatever as to the nature of disease.

Knowing that it is beyond the power of finite man to solve the insolvable he contents himself with what can be known, and bends every effort toward curing his patient, without theorizing about the unknowable. He does know that disease is a condition, an imponderable, and that all that is manifest and comprehensible are the symptoms which are indicative of a departure from the normal state. With these as his guide and with the law of the similars and with remedies so prepared that they contain nothing noxious he is able to grapple successfully with any diseased condition, and to cure any curable case, and he does not fear to assert that almost all cases of acute disease occurring in those who have led a rational, sober life are curable—provided they have not been previously maltreated with crude drugs. This assertion is borne out by the experience of hundreds of honest men, who have conscientiously adhered to the law of Homœopathy in treating sickness, and whose testimony cannot be successfully impeached. In all epidemics which have occurred during the present century this has been the case. Cholera, typhoid fever, small-pox, diphtheria, spotted fever, scarlet fever, and the late pandemic "la grippe," have been met, and in all places where Homœopathy had any chance to show its merits it came off with flying colors.

In 1855 (this is an old story but it will bear repetition), it was shown that in the cholera epidemic which had ravaged Great Britain, in the London Homœopathic Hospital the mortality was 16.4 per cent., while under allopathic treatment in the same epidemic the mortality was 59.2 per cent. In a letter on the subject Dr. McLoughlin (an allopath), Government Inspector of Hospitals, said: "You are aware that I went to your hospital prepossessed against the homœopathic system; that you had in me, in your camp, an enemy, rather than a friend. * * * That there may be no misapprehension about the cases I saw in your hospital, I will add that all I saw were true cases of cholera, in the various stages of the disease; and that I saw several cases which did well under your treatment, which I have no hesitation in saying would have sunk under any other.

"In conclusion, I must repeat to you what I have already told you, and what I have told every one with whom I have conversed, that, although an allopath by principle, education, and practice, yet, were it the will of Providence to afflict me with cholera, and to deprive me of the power to prescribe for myself, I would rather be in the hands of a homœopathic than an allopathic prescriber."

Dr. Rubini, of Naples, in an epidemic of cholera in that city several years ago, had a mortality of less than one per cent., and he treated several hundred cases.

Several years ago a young man, who had just been graduated from a homœopathic college, went to his home in the Lehigh valley and found existing an epidemic of spotted fever. In his town there are only allopathic physicians, and their mortality in that epidemic was about 100 per cent.

Hardly one case survived under their treatment. Although the people were not familiar with Homœopathy, they felt that it could do no worse than allopathy, and on trying this fledgeling Homœopathy they realized, to their astonishment, how much superior it was in results. The homœopathician's mortality was about 10 per cent. They were mostly unlettered people, but they were not so blinded by prejudice that they were not able to see the difference between old-school, death-dealing drugging, and life-saving Homœopathy.

Homœopathy, genuine Homœopathy, needs only to be put to the proof!

